# Prizes Offered by the Lexington Medical Society—Prize for the Best Thesis

**Published:** 1847-06

**Authors:** 


*Prizes offered by the Lexington Medical Society—Prize for the best Thesis.*—The Lexington Medical Society resolved to offer a prize of fifty dollars, or a gold medal or piece of plate of that value, at the option of the successful competitor, for the best Thesis submitted for the Degree of Doctor of Medicine, in the Medical Department of Transylvania University, for the session of 1847–48. Those competing for this prize are at liberty to select the subject of the Thesis.

*Annual prize.*—The Society also resolved to offer an *annual* prize of fifty dollars, or a gold medal or piece of plate of that value, for the best original essay, on a subject to be selected by a committee. In accordance with the above resolutions, the committee propose, for

1847, a prize *for the best account of Continued Fever*, as it prevails in any of the States out of New England.

*Remarks.*—The Continued Fever of the Eastern States has been very carefully studied and very fully described. It is found to correspond exactly to the *typhoid fever* of France ; and it is by that name that the disease is now generally called. The same disease is known to prevail extensively in some of the Middle, Western, and South-Western States ; but throughout these regions it has not yet been very fully or thoroughly studied. This prize is offered as one means of remedying this defect.

Other things being equal, the prize will be awarded to that essay which contains an authentic and complete account and analysis of the largest number of cases of the disease ; its leading phenomena during life, with the lesions in fatal cases ; its average and extreme duration ; the sex and age, and race, of its subjects ; the season of its prevalence ; and the prominent points of difference between it and periodical fever. It is very desirable that a careful examination should be made of the mucous membrane of the lower portion of the small intestines. Speculations about the remote and proximate causes of the disease will not add anything to the value of the essays that may be offered. The description of the disease must be sufficiently full and minute, to enable the committee to judge of the accurary of the diagnosis.

The committee also propose, for 1848, a prize *for the best account of the several forms of Periodical or Malarious Fever in the United States.*

*Remarks.*—This subject has already attracted so large a share of attention amongst physicians of the South and West, that some of them may be surprised at its being thus brought forward. But, extensively as malarious fever has been written about, there are many points of its natural history which need farther elucidation. Amongst these may be mentioned, particularly, the following :—the comparative liability of the sexes, of the black and white races, and of different periods of life, to the several forms of the disease ; the influence of race upon its severity and danger ; the relative proportions in different years and localities, of the three principal forms—intermittent, remittent and congestive ; the most common type of the pure intermittent form ; and the variations in the general character of the disease, in different seasons.

The essays must be sent, post-paid, to the Corresponding Secretary, Professor J. M. Bush, on or before the first day of January, 1848, for the first, and 1849 for second prize.

Each essay must be accompanied with a sealed address of the author. That of the successful competitor only will be opened. The essays will be considered as the property of the Society, to be deposited in their archives, or published in their transactions, as they may deem best.

The committee may suggest, that a convenient opportunity for forwarding essays from a distance, will be presented by the annual assembling of the pupils of Transylvania University, in the fall.—*Ibid.*

